# Trends and correlates of hardcore smoking in India: findings from the Global Adult Tobacco Surveys 1 & 2

**DOI:** 10.12688/wellcomeopenres.17465.2

**Published:** 2022-02-17

**Authors:** Kattiyeri Puthenveedu Veena, Elezebeth Mathews, Prakash Babu Kodali, Kavumpurathu Raman Thankappan

**Affiliations:** 1 Department of Public Health and Community Medicine, Central University of Kerala, Kasaragod, Kerala, 671315, India

**Keywords:** Hardcore smokers, Socioeconomic position, tobacco control, India

## Abstract

**Background:** Data on prevalence of hardcore smoking (HCS) among different socioeconomic status (SES) groups in low- and middle-income countries are limited. We looked at the prevalence and pattern of HCS in India with the following objectives: 1) to analyse the association between SES and HCS, 2) to find trends in HCS in different SES groups and 3) to find state-wide variations in hardcore smoking.

**Methods**: Data of individuals aged ≥25 years from the Global Adult Tobacco Survey (GATS) India 2009-10 (N= 9223) and 2016-17 (N= 7647) were used for this study. If an individual met all the following criteria: (1) current smoker, (2) smokes 10 or more cigarettes/day, (3) smokes first puff within 30 minutes after waking up, (4) no quit attempt in last 12 months, (5) no intention to quit at all or in the next 12 months, (6) lack of knowledge of harmful effect of smoking, s/he was identified as a hardcore smoker. Multiple regression analysis was done to find the factors associated with HCS.

**Results**: Prevalence of HCS deceased from 2.5% in GATS 1 to 1.9% in GATS 2: males from 6.2% to 3.9% and females from 0.3% to 0.2%. Compared to the richest group the poorest, poor and those who belonged to the middle-income group were more likely to report HCS in GATS 1 and 2. However, only in the poorest SES group there was an increase in the proportion of hardcore smokers in GATS 2 compared to GATS 1. Other factors that were significantly associated with HCS in both surveys were male gender, working adults, those with lower education, early initiation of smoking and households without any rules for smoking inside the home.

**Conclusions**: Tobacco control and cessation efforts need to focus on individuals of poor SES groups particularly in the high prevalence Indian states.

## Introduction

There were more than one billion smokers globally in the year 2019 consuming 7.4 trillion cigarettes ^1^. Smoking was attributed to 7.7 million deaths and 200 million disability adjusted life years (DALYs) and was the leading risk factor for death among males in that year ^1^. Smoking prevalence in India decreased from 13.9% to 10.7% (males 24.3% to 19.0%, females 2.9% to 1.9%) during the period 2010 to 2017 ^2^. Despite this decrease, there were 130.9 million smokers (116 million males and 14.9 million females) in India in 2019 ^1^. In 2019, total deaths attributed to smoking in India were 1.01 million (845,000 males,169,000 females) which was 10.8% of total deaths; 16.9% among male deaths and 3.85% of female deaths ^1^.

There is an argument that when smoking prevalence decreases a greater proportion of the remaining smokers are likely to be hardcore smokers (HCS). However, the recent editorial in the journal Tobacco Control stated that this argument is not supported by evidence. As per this editorial, softening (when smoking prevalence decreases, a greater proportion of the remaining smokers are likely to be non-HCS) was happening instead of hardening ^3^. In high income countries, most evidence point towards softening rather than hardening ^4, 5^. Although the definition of hardcore smoking varies across different studies, the common hardening indicators include measures of nicotine dependence and quitting behavior such as past experience of quitting and intention to quit. Even in low- and middle-income countries, a recent study reported a reduction of hard-core smokers in seven of the 10 countries included in the study and no change in one country (Mexico) ^2^. In the remaining two countries (China and Turkey) hardcore smoking increased. In India, hardcore smoking decreased from 2.5% to 1.6% as per the above study ^2^. Another study from low- and middle-income countries reported that the proportion of hardcore smokers varies from country to country and comprehensive and efficient implementation of the current tobacco control policies are likely to reduce the number of hardcore smokers ^6^. A recent systematic review on hardcore smoking argued that there was no evidence for the occurrence of hardening (i.e. when smoking prevalence decreases, the remaining smokers likely to be HCS), and tobacco control policies based on hardening should be challenged ^7^. A study from Australia reported that there was a greater accumulation of hardcore smokers in the low socioeconomic status group compared to the high socioeconomic status group indicating that hardcore smokers in low SES groups are less likely to quit ^8^. Data on the prevalence of hardcore smokers among different socioeconomic status groups in low- and middle-income countries are limited. In order to address this issue, we looked at the change in hardcore smoking during the period from 2010 to 2017 using the Global Adult Tobacco Survey (GATS) data in India with the following objectives: 1) to look at the association between socioeconomic status (SES) and HCS in India. 2) to find out the trend in HCS in different SES groups in India and 3) to find state-wide variations in hardcore smoking in India.

## Methods

### Survey

The Global Adult Tobacco Survey (GATS) monitors tobacco use (smoking and smokeless) among adults and traces key tobacco control indicators consistently in GATS countries. The GATS is a cross-sectional household survey publicly available from the Centers for Disease Control and Prevention (CDC). In India, the initial round of GATS was conducted in 2009–10, which included all 29 states and a couple of Union Territories of Chandigarh and Pondicherry, forming about 99.9% of the Indian population. The second round was implemented in 2016–17 (GATS 2) in all 30 states of India and two Union territories (UTs). In GATS 1, 69,296 individuals, aged 15 years and above, were interviewed, among which 33,767 were males and 35,529 were females. In GATS 2 a total of 74,037 individuals aged 15 years and above were interviewed including 33,772 males and 40,265 females.

### Study design and participants

GATS India was the first nationally representative survey in which electronic handheld devices were used as survey instruments. A stratified multistage probability sampling technique was used in GATS and a standardized questionnaire was used for the survey. Households were selected randomly from selected locations and within household eligible persons were interviewed randomly. Privacy was maintained during the interview. The survey method and survey instrument are described in detail elsewhere ^9^. To identify the hardcore smokers and factors associated with hardcore smoking in India, GATS India survey data of 2009–10 and 2016–17 were used. The data for GATS 1 & 2 surveys in India were accessed from CDC’s GATS survey data webpage. We included smokers aged 25 years and above because majority of the studies on hardcore smoking included smokers aged 25 years and above and, in many studies, hardcore smoking definition included at least 5-6 years of smoking history ^7^. Data from individuals aged 25 years and above (GATS 1= 56006, GATS 2= 60837) from both GATS India surveys were extracted to be included in the study ^10^.

### Variables in the study

Hardcore smoking was the outcome variable, and it was defined using several indicators based on previous studies ^11, 12^. An individual was categorized as a Hardcore smoker based on following criteria: (1) current smoker, (2) smokes 10 or more cigarettes/day, (3) smokes first puff within 30 minutes after waking up, (4) no quit attempt in last 12 months, (5) no intention to quit at all or in the next 12 months, (6) lack of knowledge of harmful effect of smoking. Several other researchers have used all six criteria or a few out of the six to define hardcore smoking ^11, 12^. All six indicators are captured as individual variables in GATS 1 & 2 datasets. In our study, we considered hardcore smoking if the individuals met all six criteria. Interviewees who answered ‘yes’ to the question that smoking causes serious illnesses (e.g., heart attack, lung cancer, stroke) were considered as knowing the harmful effect of smoking. Those who answered ‘no’ or ‘don’t know’ were defined as not having knowledge on harmful effects of smoking.

The other variables included in the analyses were: gender (male or female), type of residence (urban or rural), marital status (separated/divorced, single, married, widowed), occupation (not working, working), education (no formal schooling, education up to higher secondary, graduation and above), age group in years (25–35, 36–45, > 45years), caste (scheduled caste, scheduled tribe, other backward caste, others), age of smoking initiation (<20 years, ≥20 years), smoking practices inside home (no rules, allowed, not allowed but exceptions, never allowed) and wealth index. The wealth index was calculated as a composite index of household’s ownership of assets such as television, radio, refrigerator, fixed telephone, cell phone, flush toilet, car, scooter/moped/motorcycle, washing machine, computer/laptop etc ^13^. Based on wealth index the sample was divided into five quintiles (poorest, poor, middle, rich and richest) ^14^. As self-reported income is not a reliable indicator, wealth index calculated based on asset ownership is used as a proxy indicator for economic status ^15^.

### Statistical analysis

IBM SPSS Statistics for Windows, Version 25.0 Armonk, NY: IBM Corp was used for data cleaning, preparation, and analysis. State-wise prevalence of current daily smokers and hardcore smokers was calculated from GATS 1 and 2 datasets using univariate analysis. Country sample weights were applied to balance for the complex sampling design adopted in GATS, to approximate prevalence rates and 95% confidence intervals (95% CI). Percentage distribution of hardcore and non-hardcore smokers among the survey population was analyzed using frequency tables. Binary logistic regression analysis was done to find the factors associated with hardcore smoking. The significance level in this study was fixed at p-value<0.05

## Results

The sample characteristics for the first and second rounds of the GATS in India are described in Table 1. The proportion of adults aged more than 45 years was more in GATS 2 compared to GATS 1. Marital status and caste data were available only in GATS 2.

**Table 1.** Characteristics of individuals aged 25 and above included in the study.

Variables	GATS-1			GATS-2		
	Male (%)	Female (%)	Total (%)	Male (%)	Female (%)	Total (%)
**Age group**						
25–35 years	11425(20.4)	11369(20.3)	22794(40.7)	11559(19.0)	11194(18.4)	22753(37.4)
36–45 years	7561(13.5)	6721(12.0)	14282(25.5)	7605(12.5)	7604(12.5)	15209(25.0)
>45 years	9689(17.3)	9241(16.5)	18930(33.8)	11620(19.1)	11255(18.5)	22875(37.6)
**Residence**						
Urban	8737(15.6)	7897(14.1)	16634(29.7)	11011(18.1)	10464(17.2)	21475(35.3)
Rural	19938(35.6)	19434 (34.7)	39372(70.3)	19712(32.4)	19650(32.3)	39362(64.7)
**Marital status**						
Married	No data available			27194(44.7)	24700(40.6)	51894(85.3)
Single				2677(4.4)	669 (1.1)	3346 (5.5)
Separated/Divorced				183(0.3)	425(0.7)	608(1.0)
Widowed				730(1.2)	4259(7.0)	4989 (8.2)
**Occupation**						
Not working	3416(6.1)	19042(34.0)	22458(40.1)	3711(6.1)	21171(34.8)	24882(40.9)
Working	25259(45.1)	8289(14.8)	33548(59.9)	27073(44.5)	8882(14.6)	35955(59.1)
**Education**						
Graduation and above	3192(5.7)	1513(2.7)	4705(8.4)	4076(6.7)	2312(3.8)	6388(10.5)
Education up to Higher secondary	18314(32.7)	11537(20.6)	29851(53.3)	20259(33.3)	14114(23.2)	34373(56.5)
No formal schooling	7169(12.8)	14281(25.5)	21450(38.3)	6388(10.5)	13688(22.5)	20076(33.0)
**Caste**						
Others	No data available			8578(14.1)	7909(13.0)	16487(27.1)
Scheduled Caste				5718(9.4)	5780(9.5)	11498(18.9)
Scheduled Tribe				2738(4.5)	2920(4.8)	5658(9.3)
Other Backward Caste				13749(22.6)	13445(22.1)	27194(44.7)
**Smoking-related factors** *Age of daily smoking initiation (daily smokers-10680(GATS-1),8834(GATS-2))*						
< 20 years	5863(54.9)	545(5.1)	6408(60.0)	4673 (52.9)	336 (3.8)	5009(56.7)
>=20 years	3706(34.7)	566(5.3)	4272(40.0)	3366 (38.1)	459(5.2)	3825(43.3)
**Environmental factors** *Smoking practices inside home*						
Never allowed	10977(19.6)	10921(19.5)	21898(39.1)	15939(26.2)	15331(25.2)	31270(51.4)
Allowed	7729(13.8)	8787(15.6)	16466(29.4)	5840(9.6)	7362(12.1)	13202(21.7)
Not allowed but Exceptions	4705(8.4)	4424(7.9)	9129(16.3)	5292(8.7)	3529(5.8)	8821(14.5)
No Rules	5209(9.3)	3304(5.9)	8513(15.2)	3711(6.1)	3833(6.3)	7544(12.4)
**Wealth Index**						
Poorest	3305(5.8)	3360(6.0)	6665(11.8)	4563(7.5)	4381(7.3)	9004(14.8)
Poor	4425(8.0)	4256(7.6)	8681(15.6)	3224(5.3)	3164(5.2)	6388(10.5)
Middle	3808(6.8)	3808(6.8)	7616(13.6)	4441(7.3)	4441(7.3)	8882(14.6)
Rich	8625(15.4)	8009(14.3)	16634(29.7)	5475(9.0)	4927(8.0)	10342(17.0)
Richest	8513(15.2)	7897(14.1)	16410(29.3)	13384(22.0)	12837(21.1)	26221(43.1)

*The percentages are frequencies are derived after applying Sample Weights for GATS. GATS=Global Adult Tobacco Survey.

Change in current daily smoking and hardcore smoking prevalence between GATS 1 and GATS 2 in different states of India are given in Table 2. For India as a whole, current daily smoking decreased from 13.9% to 11.0% and hardcore smoking deceased from 2.5% to 1.9%. The relative reduction of hardcore smokers was 24% compared to 20.9% in current daily smokers. Current daily smoking prevalence in GATS 1 was highest in Mizoram (39.1%) and lowest in Goa (5.3%) while in GATS 2 the highest prevalence was in Mizoram (33.9%) and Meghalaya (32.4%) and the lowest in Goa (2.1%). Hardcore smoking prevalence in GATS 1 was highest in Mizoram (19.6%) and lowest in Goa (0%) whereas in GATS 2 the highest prevalence was in Meghalaya (4.9%) and the lowest in Goa (0%) and Sikkim (0%).

**Table 2.** Change in current daily smoking and hard-core smoking between GATS 1 and GATS 2.

States	Current daily smoking (%)			Hardcore Smoking (%)		
	GATS-1	GATS-2	Absolute Change	GATS-1	GATS-2	Absolute Change
J&K	25.4	23.0	-2.4	1.8	4.6	2.8
HP	19.8	14.5	-5.3	3.4	2.1	-1.3
Punjab	6.3	7.2	0.9	1.9	0.5	-1.4
Chandigarh	11.4	8.2	-3.2	4.3	1.4	-2.9
Uttarakhand	27.9	20.9	-7.0	3.8	4.0	0.2
Haryana	24.6	22.8	-1.8	6.8	3.6	-3.2
Delhi	19.3	9.6	-9.7	8.0	1.8	-6.2
Rajasthan	21.3	15.2	-6.1	7.2	2.4	-4.8
UP	17.4	14.4	-3.0	2.7	3.0	0.3
Chhattisgarh	10.0	5.1	-4.9	1.4	1.2	-0.2
MP	15.3	10.7	-4.6	1.4	1.8	0.4
WB	20.1	17.8	-2.3	6.4	4.4	-2.0
Jharkhand	3.6	7.2	3.6	0.4	0.2	-0.2
Odisha	9.7	5.7	-4.0	1.3	0.8	-0.5
Bihar	10.6	5.7	-4.9	0.9	0.9	0.0
Sikkim	20.7	10.8	-9.9	3.3	0.0	-3.3
Arunachal	25.5	17.8	-7.7	3.6	1.4	-2.2
Nagaland	31.9	10.7	-21.2	3.2	1.0	-2.2
Manipur	21.4	17.6	-3.8	3.4	2.4	-1.0
Mizoram	39.1	33.9	-5.2	19.6	4.8	-14.8
Tripura	29.9	24.4	-5.5	6.9	4.5	-2.4
Meghalaya	35.0	32.4	-2.6	13.0	4.9	-8.1
Assam	13.3	11.3	-2.0	1.5	3.0	1.5
Gujarat	12.8	8.9	-3.9	1.4	2.1	0.7
Maharashtra	6.8	3.5	-3.3	0.7	0.3	-0.4
Goa	5.3	2.1	-3.2	0.0	0.0	0.0
AP	19.3	16.5	-2.8	2.5	1.2	-1.3
Telangana		9.2			1.2	
Karnataka	13.3	8.4	-4.9	2.6	0.9	-1.7
Kerala	10.1	8.0	-2.1	1.7	1.4	-0.3
Tamil Nadu	9.0	10.9	1.9	1.9	1.7	-0.2
Puducherry	9.2	7.5	-1.7	1.3	1.2	-0.1
India	13.9	11.0	-2.9	2.5	1.9	-0.6

*The percentages are derived after applying sample weights. GATS=Global Adult Tobacco Survey.

Change in current daily smoking and hardcore smoking prevalence between GATS 1 and GATS 2 among males are given in Table 3. Current daily smoking prevalence among males in India decreased from 23.9% in GATS 1 to 19.6% in GATS 2 and that of hardcore smoking from 4.7% to 3.6%. Relative reduction of current daily smokers was 18% and that of hardcore smokers was 23.4%. Current daily smoking prevalence among males in GATS 1 was highest in Meghalaya (64.7%) and lowest in Jharkhand (6.3%) and Goa (8.2%). In GATS 2 also the highest and lowest prevalence was in the states Meghalaya (58.3%) and Goa (4.3%), albeit decreased rates in both states. In GATS 1 the highest prevalence of hardcore smoking among males was in Mizoram (37.5%) and lowest was in Goa (0%) while in GATS 2 the highest prevalence was in Meghalaya (9.9%) and lowest in Goa (0%).

**Table 3.** Change in daily smoking and Hardcore smoking between GATS 1 and GATS 2- Males.

States	Current daily smoking (%)			Hardcore Smoking (%)		
	GATS-1	GATS-2	Absolute Change	GATS-1	GATS-2	Absolute Change
J&K	40.1	38.8	-1.3	3.4	8.1	4.7
HP	36	27.7	-8.3	6.3	3.8	-2.5
Punjab	11.8	13.6	1.8	3.7	0.8	-2.9
Chandigarh	20	15.0	-5.0	7.5	2.5	-5.0
Uttarakhand	39.7	35.0	-4.7	5.8	7.3	1.5
Haryana	41.9	39.3	-2.6	12.9	6.7	-6.2
Delhi	32.7	16.4	-16.3	14.3	3.2	-11.1
Rajasthan	36.1	25.7	-10.4	13.6	4.4	-9.2
UP	27.6	24.8	-2.8	4.8	5.2	0.4
Chhattisgarh	15.4	10.4	-5.0	2.3	2.5	0.2
MP	27.4	20.0	-7.4	2.5	3.5	1.0
WB	36.7	33.7	-3.0	12	8.5	-3.5
Jharkhand	6.3	12.8	6.5	0.7	0.5	-0.2
Odisha	18.5	11.0	-7.5	2.5	1.7	-0.8
Bihar	11.9	7.1	-4.8	1	1.3	0.3
Sikkim	25	15.0	-10.0	0	0	0.0
Arunachal	39.3	28.9	-10.4	6.9	2.6	-4.3
Nagaland	47.1	20.8	-26.3	5.9	1.9	-4.0
Manipur	29.3	29.3	0.0	5.2	4.9	-0.3
Mizoram	58.3	51.6	-6.7	37.5	9.4	-28.1
Tripura	45.1	39.3	-5.8	12.1	8.1	-4.0
Meghalaya	64.7	58.3	-6.4	25	9.9	-15.1
Assam	24	21.3	-2.7	2.9	5.3	2.4
Gujarat	23.8	16.5	-7.3	2.7	3.9	1.2
Maharashtra	12.6	5.3	-7.3	1.3	0.6	-0.7
Goa	8.2	4.3	-3.9	0.0	0.0	0.0
AP	33.9	27.7	-6.2	5.2	2.4	-2.8
Telangana		17.2			2.4	
Karnataka	26.3	15.9	-10.4	5.2	1.7	-3.5
Kerala	21.4	17.2	-4.2	3.6	3.0	-0.6
Tamil Nadu	17.9	22.0	4.1	3.8	3.5	-0.3
Puduchery	18.4	13.2	-5.2	2.6	2.6	0.0
India	23.9	19.6	-4.3	4.7	3.6	-1.1

*The percentages are derived after applying sample weights. GATS= Global Adult Tobacco Survey; HCS= Hardcore smoking; J&K= Jammu and Kashmir; HP= Himachal Pradesh; UP= Uttar Pradesh; MP= Madhya Pradesh; WB= West Bengal; AP= Andhra Pradesh

Change in current daily smoking and hard-core smoking prevalence between GATS 1 and GATS 2 among females is given in Table 4. Current daily smoking prevalence among females in India decreased from 3.3% in GATS 1 to 2.2% in GATS 2. The hardcore smoking among females aged 25 years and above at the national level remained unchanged standing at 0.2% to 0.2%. Relative reduction was 33% for current daily smokers. Current daily smoking prevalence among females in GATS 1 was highest in Mizoram (19.0%) and lowest in states like Kerala (0.0%), Maharashtra (0.0%) and Puducherry (0.0%). In GATS 2 the highest prevalence of current daily smoking was in Mizoram (12.9%) and lowest prevalence of 0.0% was reported from Chandigarh. In GATS 1 the highest prevalence of hardcore smoking among females was in Mizoram (4.3%) and hardcore smoking was not reported from 20 states and union territories, while in GATS 2 the highest prevalence was in Mizoram (3.2%) and hardcore smoking was not reported from 18 states and union territories.

**Table 4.** Change in current daily smoking and hard-core smoking between GATS 1 and GATS 2 – Females.

States	Current daily smoking (%)			Hardcore Smoking (%)		
	GATS-1	GATS-2	Absolute Change	GATS-1	GATS-2	Absolute Change
J&K	8.9	5.4	-3.5	0.0	0.6	0.6
HP	4.0	1.9	-2.1	0.6	0.5	-0.1
Punjab	0.4	0.4	0.0	0.0	0.1	0.1
Chandigarh	0.0	0.0	0.0	0.0	0.0	0.0
Uttarakhand	5.0	7.1	2.1	0.0	0.7	0.7
Haryana	5.5	5.3	-0.2	0.0	0.1	0.1
Delhi	2.6	2.1	-0.5	0.0	0.2	0.2
Rajasthan	5.9	4.2	-1.7	0.6	0.2	-0.4
UP	6.3	3.6	-2.7	0.4	0.6	0.2
Chhattisgarh	4.1	0.1	-4.0	0.4	0.0	-0.4
MP	2.4	1.0	-1.4	0.2	0.0	-0.2
WB	2.4	1.1	-1.3	0.4	0.1	-0.3
Jharkhand	0.9	1.5	0.6	0.0	0.0	0.0
Odisha	1.0	0.1	-0.9	0.1	0.0	-0.1
Bihar	9.3	4.3	-5.0	0.8	0.5	-0.3
Sikkim	14.3	5.9	-8.4	0.0	0.0	0.0
Arunachal	11.1	5.7	-5.4	0.0	0.0	0.0
Nagaland	13.3	0.0	-13.3	0.0	0.0	0.0
Manipur	13.8	6.0	-7.8	1.7	0.0	-1.7
Mizoram	19.0	12.9	-6.1	4.3	3.2	-1.1
Tripura	13.3	9.9	-3.4	1.2	0.9	-0.3
Meghalaya	4.1	5.7	1.6	0.0	0.0	0.0
Assam	1.6	1.0	-0.6	0.0	0.6	0.6
Gujarat	1.1	0.7	-0.4	0.0	0.1	0.1
Maharashtra	0.0	1.6	1.6	0.0	0.0	0.0
Goa	0.0	0.0	0.0	0.0	0.0	0.0
AP	5.3	5.8	0.5	0.0	0.0	0.0
Telangana		1.7			0.0	
Karnataka	0.3	0.9	0.6	0.0	0.0	0.0
Kerala	0.0	0.3	0.3	0.0	0.0	0.0
Tamil Nadu	0.1	0.1	0.0	0.0	0.0	0.0
Puduchery	0.0	0.0	0.0	0.0	0.0	0.0
India	3.3	2.2	-1.1	0.2	0.2	0.0

*The percentages are derived after applying sample weights. GATS= Global Adult Tobacco Survey; HCS= Hardcore smoking; J&K= Jammu and Kashmir; HP= Himachal Pradesh; UP= Uttar Pradesh; MP= Madhya Pradesh; WB= West Bengal; AP= Andhra Pradesh

Factors associated with hardcore smoking in India based on regression analysis of GATS 1 data are presented in Table 5. Males, rural residents, working adults and those without any formal schooling were more likely to report hardcore smoking compared to their counterparts. Compared to those aged 25–35 years, those aged 36–45 years were more likely to report hardcore smoking. Compared to the richest group as per the wealth index all other categories were more likely to report hardcore smoking. Adults who initiated smoking below 20 years were more likely to report hardcore smoking compared to those who initiated after 20 years. With regard to smoking practices inside the home, those who did not have any rules and those allowed smoking inside the home were more likely to report hardcore smoking.

**Table 5.** Factors associated with HCS in GATS 1: Results of Binary logistic regression analysis (N= 56006).

Factors	Hard-core smokers (%)	Unadjusted OR (95% CI)	Adjusted OR (95% CI)	p value
**1. Socio-demographic characteristics** *A. Gender*				
Female Male	0.2 4.7	Reference 2.83(2.19-3.66)	Reference 2.57(1.92-3.45)	<0.001
*B. Type of residence*				
Urban Rural	1.9 2.8	Reference 1.04(0.91-1.19)	Reference 1.23(1.06-1.44)	0.008
*C. Occupation*				
Not working Working	0.8 3.7	Reference 1.68(1.42-1.99)	Reference 1.37(1.12-1.67)	0.002
*D. Education*				
Graduation and above Education up to Higher secondary No formal schooling	0.8 2.6 2.7	Reference 2.02(1.45-2.83) 2.11(1.51-2.97)	Reference 1.33(0.92-1.92) 1.34(0.91-1.96)	0.129 0.134
*E. Age group in years*				
25–35 years 36–45 years 45 years and above	1.5 3.1 3.2	Reference 1.45(1.25-1.68) 1.33(1.16-1.54)	Reference 1.30(1.11-1.54) 1.35(1.15-1.57)	0.001 <0.001
*F. Wealth index*				
Richest Poorest Poor Middle Rich	1.4 2.7 3.2 3.4 2.8	Reference 1.29(1.05-1.59) 1.55(1.29-1.87) 1.85(1.53-2.24) 1.58(1.34-1.87)	Reference 1.29(1.01-1.65) 1.51(1.21-1.89) 1.86(1.49-2.32) 1.51(1.24-1.82)	0.043 <0.001 <0.001 <0.001
**2. Smoking related factors (n=10680)**				
*Age of smoking initiation* 20 years and above Below 20 years	15.2 20.3	Reference 1.42(1.26-1.61)	Reference 1.35(1.19-1.54)	<0.001
**3. Environmental factors**				
A. Second-hand smoke exposure 1. *Smoking practices inside home*				
Never allowed Allowed Not allowed but exceptions No Rules	0.4 5.9 1.0 2.9	Reference 4.16(3.29-5.27) 1.61(1.84-2.19) 2.52(1.94-3.27)	Reference 2.86(2.23-3.68) 1.53(1.11-2.12) 2.09(1.58-2.76)	<0.001 0.010 <0.001

GATS= Global Adult Tobacco Survey; HCS= Hardcore smoking; OR= odds ratio; CI= confidence interval.

Factors associated with hardcore smoking in India based on regression analysis of GATS 2 data are presented in Table 6. Males, working adults and those without any formal schooling were more likely to report hardcore smoking compared to their counterparts similar to GATS 1. However, the rural urban difference in hardcore smoking was not significant in GATS 2. With regard to the age group, those who were in the age group of more than 45 years reported hardcore smoking more than those in the age group of 25–35 years. Regarding the wealth index categories, those who belonged to the poorest, poor and the middle group categories were more likely to report hardcore smoking compared to the richest wealth index group. Adults who initiated smoking below 20 years were more likely to report hardcore smoking compared to those who initiated after 20 years. With regard to smoking practices inside the home, those who did not have any rules and those who allowed smoking inside their home were more likely to report hardcore smoking similar to GATS 1.

**Table 6.** Factors associated with HCS among individuals aged 25 years and above in GATS 2 (N=60837).

Factors	Hardcore smokers (%)	Unadjusted OR (95% CI)	Adjusted OR (95% CI)	p value
**1.Socio-demographic Characteristics** *A. Gender*				
Female Male	0.2 3.6	Reference 2.27(1.75-2.96)	Reference 3.04(2.19-4.22)	<0.001
*B. Type of residence*				
Urban Rural	1.3 2.2	Reference 1.19(1.03-1.36)	Reference 1.16(0.98-1.35)	0.073
*C. Occupation*				
Not working Working	0.6 2.8	Reference 1.25(1.06-1.49)	Reference 1.22(1.01-1.22)	0.050
*D. Education*				
Graduation and above Education up to Higher secondary No formal schooling	0.3 1.9 2.4	Reference 2.86(1.86-4.41) 3.54(2.29-5.46)	Reference 1.76(1.12-2.77) 1.94(1.22-3.08)	0.014 0.005
*E. Age group in years*				
25–35 years 36–45 years 45 years and above	1.0 1.8 2.9	Reference 1.26(1.06-1.50) 1.67(1.44-1.95)	Reference 1.11(0.92-1.34) 1.54(1.31-1.83)	0.261 <0.001
F. *Wealth index*				
Richest Poorest Poor Middle Rich	1.2 3.0 2.7 2.6 1.8	Reference 1.45(1.23-1.72) 1.42(1.17-1.72) 1.48(1.24-1.76) 1.17(0.97-1.41)	Reference 1.39(1.14-1.69) 1.36(1.10-1.69) 1.31(1.08-1.58) 1.12(0.92-1.37)	0.001 0.005 0.006 0.241
**2.Smoking related factors** (n=8834)				
*Age of smoking initiation* 20 years and above Below 20 years	14.7 19.4	Reference 1.39(1.23-1.58)	Reference 1.33(1.17-1.52)	<0.001
**3.Environmental factors**				
A. Second-hand smoke exposure 1. *Smoking practices inside home*				
Never allowed Allowed Not allowed but exceptions No Rules	0.5 5.4 1.3 2.5	Reference 2.87(2.40-3.43) 1.08(0.85-1.38) 2.21(1.78-2.75)	Reference 2.26(1.87-2.72) 0.98(0.76-1.25) 1.92(1.52-2.41)	<0.001 0.849 <0.001

GATS= Global Adult Tobacco Survey; HCS= Hardcore smoking; OR= odds ratio; CI= confidence interval.

**Figure 1.**
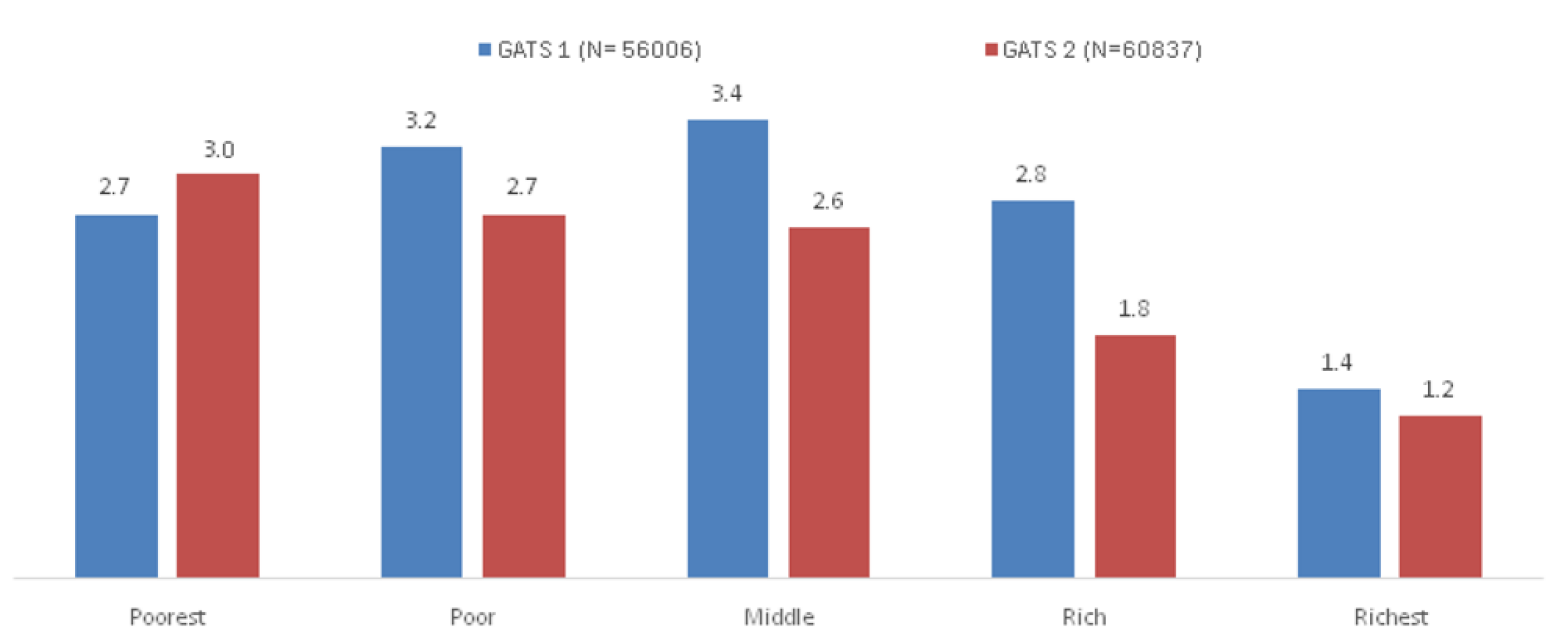
Percentage of hardcore smokers across wealth quintiles among individuals aged 25 years and above in GATS 1 and GATS 2. *The percentages are derived after applying sample weights. GATS= Global Adult Tobacco Survey.

The percentage of hardcore smokers across wealth quintiles among individuals aged 25 years and above in GATS 1 and GATS 2 is given in Figure 1. Only in the poorest wealth quintile was there an increase in the proportion of hardcore smokers in GATS 2 compared to GATS 1.

The proportion of hardcore smokers among current daily smokers and the changes between GATS 1 and GATS 2 are given in Table 7. There was an overall reduction of 0.8% in the percentage of hardcore smokers among current daily smokers in India in GATS 2 compared to GATS 1. In 11 states and union territories there was increase in the proportion of hardcore smokers ranging from 0.5% in Kerala to 15% in Assam. In all other states and union territories there was decrease in the proportion of hardcore smoking. In Goa the proportion of hardcore smokers remained zero in both GATS 1 and 2. 

**Table 7.** Change in proportion of hardcore smokers among current daily smokers aged >= 25 years between GATS-1 and GATS-2.

States	GATS-1		GATS-2		% Change in Hardcore smokers among current daily smokers from GATS 1 to GATS 2
	% of Current Daily Smokers among adults ≥ 25 years	% of Hardcore smokers among current daily smokers	% of Current Daily Smokers among adults ≥ 25 years	% of Hardcore smokers among current daily smokers	
J&K	25.4	7.2	23	20	12.8
HP	19.8	17.4	14.5	14.5	-2.9
Punjab	6.3	30.3	7.2	6.5	-23.8
Chandigarh	11.4	37.5	8.2	14.3	-23.2
Uttarakhand	27.9	13.7	20.9	19	5.3
Haryana	24.6	27.6	22.8	15.7	-11.9
Delhi	19.3	41.2	9.6	18.5	-22.7
Rajasthan	21.3	33.8	15.2	15.7	-18.1
UP	17.4	15.6	14.4	20.5	4.9
Chhattisgarh	10	14.1	5.1	23.9	9.8
MP	15.3	9.3	10.7	16.4	7.1
WB	20.1	31.8	17.8	24.9	-6.9
Jharkhand	3.6	10.4	7.2	3.2	-7.2
Odisha	9.7	13.5	5.7	14.8	1.3
Bihar	10.6	8.5	5.7	16.1	7.6
Sikkim	20.7	14.3	10.8	0.0	-14.3
Arunachal	25.5	14.3	17.8	7.7	-6.6
Nagaland	31.9	9.7	10.7	9.1	-0.6
Manipur	21.4	16	17.6	13.8	-2.2
Mizoram	39.1	50	33.9	15.0	-35
Tripura	29.9	23.1	24.4	18.5	-4.6
Meghalaya	35	37.1	32.4	15.2	-21.9
Assam	13.3	11.3	11.3	26.3	15
Gujarat	12.8	10.7	8.9	23.4	12.7
Maharashtra	6.8	10.1	3.5	8.7	-1.4
Goa	5.3	0.0	2.1	0.0	0
AP	19.3	13.1	16.5	7.2	-5.9
Telangana	NA	NA	9.2	12.8	NA
Karnataka	13.3	19.7	8.4	10.2	-9.5
Kerala	10.1	16.7	8	17.2	0.5
Tamil Nadu	9	21.`12	10.9	15.8	-5.4
Puducherry	9.2	12.5	7.5	16.7	4.2
India	13.9	18	11	17.2	-0.8

*The percentages are derived after applying sample weights. GATS= Global Adult Tobacco Survey; HCS= Hardcore smoking; J&K= Jammu and Kashmir; HP= Himachal Pradesh; UP= Uttar Pradesh; MP= Madhya Pradesh; WB= West Bengal; AP= Andhra Pradesh

## Discussion

There were reductions in the prevalence of current daily smoking and hardcore smoking in India during the period between GATS 1 and GATS 2. A relative reduction of hardcore smoking prevalence of 24% was more than the 20.9% reduction in current daily smoking indicating softening of smoking rather than hardening during the period from GATS 1 to GATS 2. Compared to the GATS 1 data hardcore smoking increased only in the poorest wealth group in GATS 2 while in all the other wealth groups, hardcore smoking decreased demonstrating a greater accumulation of hardcore smokers in the poorest group as reported from Australia ^8^. Current smoking prevalence was also reported to be highest among the poorest groups in India ^14^. While the proportion of hardcore smokers in the poorest group remained high after a period of seven years between GATS 1 and GATS 2, in all the other wealth groups, there was a decline. Tobacco control measures need to focus on people belonging to the poorest group and offer them help to quit tobacco since they are likely to be more resistant to comply as reported recently in a US study ^16^.

In GATS 1 the association between hardcore smoking and the wealth groups did not show a gradient. Compared to the richest wealth group those belonging to the middle wealth group showed the highest odds of reporting hardcore smoking whereas in GATS 2 the odds of reporting hardcore smoking increased with decreasing wealth index. The accumulation of hardcore smokers was gradually shifting to the poorest group in GATS 2. While there was an overall decrease in hardcore smoking some population groups such as those belonging to the poorest section of the society seem to continue hardcore smoking. It may be important to initiate tailor made tobacco cessation efforts for smokers in the low SES groups. 

While majority of the Indian states reported reduction in hardcore smokers a few states such as Jammu and Kashmir, UP, Bihar, Chhattisgarh, MP, Uttarakhand, Assam and Gujarat reported an increase in the prevalence of hardcore smokers. Respondents from the state of Goa did not report hardcore smoking in both the surveys, and there was a reduction in the current daily smoking prevalence. This highlights the importance of implementation of Cigarettes and Other Tobacco Products Act (COTPA) in all states particularly those where there was increase in hardcore smoking. In the states where there was an increase in hardcore smoking there was reduction in current daily smoking during the same period. In Odisha and Kerala, interesting findings were observed. Both the states had an overall reduction in the current smokers and hardcore smokers as a percentage among total sample ≥ 25 years. However, the percentage of hardcore smokers within current smokers from GATS 1 to GATS 2 has grown in Odisha and Kerala with a relative increase of 9.6% and 3.0%, respectively. This could be due to the accumulation of hardcore smokers when the overall prevalence of smoking reduced as reported in the Australian study ^8^. Only three states reported an increase in current daily smoking: Punjab, Jharkhand and Tamil Nadu. In all these three states there was reduction in hardcore smoking.

Since smoking in India and most south Asian countries is predominantly a male behavior it is important to see the trends in hardcore smoking among males. Among the states where there was increase in hardcore smoking in the total population, Jammu & Kashmir, Chhattisgarh, Uttar Pradesh and Bihar etc., also reported an increase in hardcore smoking among males, although the increase was small. In Puducherry there was no change in hardcore smoking among males during this period.

Hardcore smoking was not reported by females in several states in GATS 2. However, a few states namely Jammu and Kashmir, Punjab, Uttarakhand, Haryana, Delhi, UP, Assam, and Gujarat reported an increase in hardcore smoking indicating an unhealthy hardcore smoking trend in women. The increasing trend in smoking among women in India was reported earlier also ^17^. This could be due to the targeting of women and people belonging to low socioeconomic group by the tobacco companies circumventing the tobacco control laws in India as reported earlier ^18^.

In GATS 1 the major factors associated with hardcore smoking were male gender, rural residents, working adults, older adults, those who initiated smoking before 20 years and those with no schooling or low education. Significance of urban rural difference of hardcore smoking disappeared in GATS 2. This was because of the reduced prevalence of hardcore smoking in both rural and urban areas in GATS 2 compared to the GATS 1. Those who initiated smoking before 20 years were more likely to report hardcore smoking in both GATS 1 and GATS 2 which was in the expected lines since early initiation has been reported to result in tobacco addiction more often that later initiation of smoking ^19^.

Regarding smoking practices inside home, in both GATS 1 and GATS 2 hardcore smoking was more likely to be reported where smoking was allowed inside home, where there were no rules for smoking inside home or there were exemptions for the rule compared to those households where smoking was not allowed inside the home. This is a very positive finding that can be replicated in several Indian and other similar settings. It was already reported that such smoke free home initiatives of not allowing smoking inside home in Kerala State of India ^20^ and Indonesia ^21^ were successfully implemented.

Although the use of the term hardcore smoking and hardening has been challenged recently ^22^ researchers continue to report hardcore smoking and the groups in which this continues to be a problem. The strength of this study was the large representative data of the entire country. A limitation was that tobacco use was self-reported and there was no validation by bio-chemical measures such as cotinine estimation.

## Conclusions

This study based on GATS 1 and GATS 2 data found that there was reduction in current daily smoking and hardcore smoking in India during the period between the two surveys. Although there was reduction in current daily smoking and hardcore smoking in the country as a whole a few states namely Punjab, Jharkhand and Tamil Nadu reported an increase in current daily smoking and six states namely Jammu and Kashmir, UP, MP, Uttarakhand, Assam and Gujarat reported an increase in hardcore smoking. In all these six states where there was increase in hardcore smoking, current daily smoking prevalence decreased probably due to the accumulation of hardcore smokers as a result of reduced prevalence of current daily smokers. It was also seen that only in the poorest wealth group there was an increase in the hardcore smoking during the period between GATS 1 and GATS 2. In all other wealth groups, there was reduction in hardcore smoking. This is similar to the finding of the study from Australia that when current smoking prevalence decreases there is likely to be an accumulation of hardcore smokers particularly in the poorest socioeconomic groups. Tobacco control and cessation measures need to be implemented focusing on people belonging to the poorest socioeconomic groups in India.

## Data availability

### Underlying data

Open Science Framework: Trends and Correlates of Hardcore Smoking in India- Findings from Global Adult Tobacco Survey 1 & 2. https://doi.org/10.17605/OSF.IO/FRBQ5 ^10^.

This project contains the following underlying data:

- Global Adult Tobacco Survey 1 data (extracted and cleaned as per study objective)
- Global Adult Tobacco Survey 1 code book
- Global Adult Tobacco Survey 2 data (extracted and cleaned as per study objective)
- Global Adult Tobacco Survey 2 code book

Data description: The dataset comprises the data from Round 1 and Round 2 of Global Adult Tobacco Survey (GATS) conducted in India. The data sets comprise the variables which were extracted and cleaned as per the objective of the study. The original data sets of GATS surveys are available in the public domain and are accessible at https://nccd.cdc.gov/GTSSDataSurveyResources/Ancillary/DataReports.aspx?CAID=2.

The data set includes the following variables:

1. Sample weights of the data
2. Variables on socio-demographic characteristics of the participants (gender, age, residence, educational status, state, occupational status etc.)
3. Variables Concerning smoking status of the participants (current smoking status, age of initiating smoking)
4. Variables concerning hardcore smoking status of the participants
5. Variables concerning wealth index of the participants.

Data are available under the terms of the Creative Commons Attribution 4.0 International license (CC-BY 4.0).
