# Supplementary material for: Trends and correlates of hardcore smoking in India: findings from the Global Adult Tobacco Surveys 1 & 2 [version 2; peer review: 1 approved]
Source: Wellcome Open Res. Author manuscript; Available in PMC 2022 Jun 10. (PMC7612836)
Supplement: Supplementary Materials [file EMS145404-supplement-Supplementary_Materials.pdf]

Open Peer Review

Current Peer Review Status: ✔

Version 2

Reviewer Report 25 February 2022

https://doi.org/10.21956/wellcomeopenres.19618.r48794

© 2022 Thekkumkara Surendran Nair A. This is an open access peer review report distributed under the terms of the Creative Commons Attribution License, which permits unrestricted use, distribution, and reproduction in any medium, provided the original work is properly cited.

✔ **Anish Thekkumkara Surendran Nair**
Community Medicine, Government Medical College Thiruvananthapuram, Thiruvananthapuram, Kerala, India

Approved.

Competing Interests: No competing interests were disclosed.

Reviewer Expertise: Epidemiology, public health and infectious disease epidemiology

I confirm that I have read this submission and believe that I have an appropriate level of expertise to confirm that it is of an acceptable scientific standard.

Version 1

Reviewer Report 21 January 2022

https://doi.org/10.21956/wellcomeopenres.19313.r47827

© 2022 Thekkumkara Surendran Nair A. This is an open access peer review report distributed under the terms of the Creative Commons Attribution License, which permits unrestricted use, distribution, and reproduction in any medium, provided the original work is properly cited.

✔ **Anish Thekkumkara Surendran Nair**
Community Medicine, Government Medical College Thiruvananthapuram, Thiruvananthapuram, Kerala, India

It is a nice analysis of GATS data to check the relationship between Socio-economic status and prevalence of hard core smoking. The quality of the paper may get better if the authors are able to

address the following issues:

1. It is not clear that why the authors took 25 years as a cut-off. "Age plays a role in hardening of smoking" is given as the reason to avoid younger age group, but it appears to be a reason for the importance of including people less than 25 also in the study. Reference number 10 given by the authors also argues the importance of young smokers in India. It is indicated in the first table of the current article that more than one-third of habitual smokers in India acquire that habit in an early age even less than 20 years. The prevalence estimations of the authors could also be incomparable to other studies because of a different age cut-off used other than that of GATS. So a stratified analysis of people less than 25 could also be added. Or it may be mentioned even in the title that the data is applicable to above 25 year old only.

2. Trend in HCC across socio-economic groups may be statistically tested as it is one of the objectives of the study.

3. Figure 3 appears to be confusing. A table may be more helpful for the readers.

4. Unadjusted risk ratios/OR could also be given in tables 5 and 6. It will help the readers to measure the confounding by external factors.

5. One of the major findings, compared to GATS 1 a socio-economic grading is noticed in prevalence of HCS (as moving from richest quintile the risk ratio increases) may be discussed. Now it may be more important to target the low SES group and targeted approaches may be initiated for people in low SES.

Is the work clearly and accurately presented and does it cite the current literature?

Yes

Is the study design appropriate and is the work technically sound?

Partly

Are sufficient details of methods and analysis provided to allow replication by others?

Yes

If applicable, is the statistical analysis and its interpretation appropriate?

Partly

Are all the source data underlying the results available to ensure full reproducibility?

Yes

Are the conclusions drawn adequately supported by the results?

Yes

Competing Interests: Dr. K. R. Thankappan is known to me. They happened to be the editor of an article to which I am one of the authors: https://www.frontiersin.org/articles/10.3389/fpubh.2021.747065/full.

Reviewer Expertise: Epidemiology, public health and infectious disease epidemiology

I confirm that I have read this submission and believe that I have an appropriate level of expertise to confirm that it is of an acceptable scientific standard.

Comments on this article

Version 1

Author Response 13 Feb 2022

Elezebeth Mathews, Central University of Kerala, Kasaragod, India

We have addressed the reviewer comments and revised the earlier version of the published manuscript. The following changes are made:

1. The following sentence is added in the revised manuscript, "We included smokers aged 25 years and above because the majority of the studies on hardcore smoking included smokers aged 25 years and above and, in many studies, the hardcore smoking definition included at least 5-6 years of smoking history[7]."

2. We have included the trend in hardcore smoking across socio-economic groups and the unadjusted odds ratio is reported (Table 5 and 6).

3. We have also added a new table (table 7) replacing Figure 2 on the change in the proportion of hardcore smokers among current daily smokers aged >= 25 years between GATS-1 and GATS-2.

4. We have added a new sentence as the last sentence in the second para of the discussion section. "It may be important to initiate tailor-made tobacco cessation efforts for smokers in the low SES groups."

Competing Interests: None
