# Supplementary material for: Trends and correlates of hardcore smoking in India: findings from the Global Adult Tobacco Surveys 1 & 2
Source: Wellcome Open Res. 2022 Jul 27;6:353. Originally published 2021 Dec 20. [Version 3] doi: 10.12688/wellcomeopenres.17465.3 (PMC7612836; doi:10.12688/wellcomeopenres.17465.3)
Supplement: Peer review history

# Peer review: Reviewer response for version 3

**DOI:** 10.21956/wellcomeopenres.20037.r51730

**Published:** 2022-08-05

**Authors:** Coral Gartner

**Affiliations:** 1 NHMRC Centre for Research Excellence on Achieving the Tobacco Endgame, School of Public Health, The University of Queensland, Brisbane, Qld, 4007, Australia

I have no further comments for the authors to address

Is the work clearly and accurately presented and does it cite the current literature?

Yes

If applicable, is the statistical analysis and its interpretation appropriate?

Yes

Are all the source data underlying the results available to ensure full reproducibility?

Yes

Is the study design appropriate and is the work technically sound?

Partly

Are the conclusions drawn adequately supported by the results?

Partly

Are sufficient details of methods and analysis provided to allow replication by others?

Yes

Reviewer Expertise:

Epidemiology, tobacco control policy

I confirm that I have read this submission and believe that I have an appropriate level of expertise to confirm that it is of an acceptable scientific standard.

# Peer review: Reviewer response for version 2

**DOI:** 10.21956/wellcomeopenres.19618.r50298

**Published:** 2022-06-13

**Authors:** Coral Gartner

**Affiliations:** 1 NHMRC Centre for Research Excellence on Achieving the Tobacco Endgame, School of Public Health, The University of Queensland, Brisbane, Qld, 4007, Australia

This study analyses two waves of the GATS to examine changes in the prevalence of Hardcore smoking over time in India.

 My main comment is related to the definition of 'Hardcore Smoking' employed in the manuscript. I don’t understand why lack of knowledge of harmful effects of smoking is considered an attribute of HCS. It could be considered more ‘hardcore’ to know that smoking is harmful but to continue regardless than through lack of awareness. I don’t think that including lack of awareness of health impacts of smoking is a useful measure of HCS. It would be good to demonstrate what the results would be if this part of the definition were omitted.

**Minor issues:**

- “Smoking was attributed to…” - I believe this should be phrased the other way around, i.e., the deaths were attributed to smoking not smoking attributed to the deaths.
- I was surprised to see no mention of smokeless tobacco in the discussion, given high rates of smokeless tobacco use in India compared to other countries mentioned in the manuscript (e.g. Australia).

Is the work clearly and accurately presented and does it cite the current literature?

Yes

If applicable, is the statistical analysis and its interpretation appropriate?

Yes

Are all the source data underlying the results available to ensure full reproducibility?

Yes

Is the study design appropriate and is the work technically sound?

Partly

Are the conclusions drawn adequately supported by the results?

Partly

Are sufficient details of methods and analysis provided to allow replication by others?

Yes

Reviewer Expertise:

Epidemiology, tobacco control policy

I confirm that I have read this submission and believe that I have an appropriate level of expertise to confirm that it is of an acceptable scientific standard, however I have significant reservations, as outlined above.

# Peer review: Reviewer response for version 2

**DOI:** 10.21956/wellcomeopenres.19618.r48794

**Published:** 2022-02-25

**Authors:** Anish Thekkumkara Surendran Nair

**Affiliations:** 1 Community Medicine, Government Medical College Thiruvananthapuram, Thiruvananthapuram, Kerala, India

Approved.

Is the work clearly and accurately presented and does it cite the current literature?

Yes

If applicable, is the statistical analysis and its interpretation appropriate?

Partly

Are all the source data underlying the results available to ensure full reproducibility?

Yes

Is the study design appropriate and is the work technically sound?

Partly

Are the conclusions drawn adequately supported by the results?

Yes

Are sufficient details of methods and analysis provided to allow replication by others?

Yes

Reviewer Expertise:

Epidemiology, public health and infectious disease epidemiology

I confirm that I have read this submission and believe that I have an appropriate level of expertise to confirm that it is of an acceptable scientific standard.

# Peer review: Reviewer response for version 1

**DOI:** 10.21956/wellcomeopenres.19313.r47827

**Published:** 2022-01-21

**Authors:** Anish Thekkumkara Surendran Nair

**Affiliations:** 1 Community Medicine, Government Medical College Thiruvananthapuram, Thiruvananthapuram, Kerala, India

It is a nice analysis of GATS data to check the relationship between Socio-economic status and prevalence of hard core smoking. The quality of the paper may get better if the authors are able to address the following issues:

1. It is not clear that why the authors took 25 years as a cut-off. "Age plays a role in hardening of smoking" is given as the reason to avoid younger age group, but it appears to be a reason for the importance of including people less than 25 also in the study. Reference number 10 given by the authors also argues the importance of young smokers in India. It is indicated in the first table of the current article that more than one-third of habitual smokers in India acquire that habit in an early age even less than 20 years. The prevalence estimations of the authors could also be incomparable to other studies because of a different age cut-off used other than that of GATS. So a stratified analysis of people less than 25 could also be added. Or it may be mentioned even in the title that the data is applicable to above 25 year old only.
2. Trend in HCC across socio-economic groups may be statistically tested as it is one of the objectives of the study.
3. Figure 3 appears to be confusing. A table may be more helpful for the readers.
4. Unadjusted risk ratios/OR could also be given in tables 5 and 6. It will help the readers to measure the confounding by external factors.
5. One of the major findings, compared to GATS 1 a socio-economic grading is noticed in prevalence of HCS (as moving from richest quintile the risk ratio increases) may be discussed. Now it may be more important to target the low SES group and targeted approaches may be initiated for people in low SES.

Is the work clearly and accurately presented and does it cite the current literature?

Yes

If applicable, is the statistical analysis and its interpretation appropriate?

Partly

Are all the source data underlying the results available to ensure full reproducibility?

Yes

Is the study design appropriate and is the work technically sound?

Partly

Are the conclusions drawn adequately supported by the results?

Yes

Are sufficient details of methods and analysis provided to allow replication by others?

Yes

Reviewer Expertise:

Epidemiology, public health and infectious disease epidemiology

I confirm that I have read this submission and believe that I have an appropriate level of expertise to confirm that it is of an acceptable scientific standard.
